# Rapid automated lumen segmentation of coronary optical coherence tomography images followed by 3D reconstruction of coronary arteries

**DOI:** 10.1117/1.JMI.11.1.014004

**Published:** 2024-01-02

**Authors:** Wei Wu, Merjulah Roby, Akshat Banga, Usama M. Oguz, Vinay Kumar Gadamidi, Charu Hasini Vasa, Shijia Zhao, Vineeth S. Dasari, Anjani Kumar Thota, Sartaj Tanweer, Changkye Lee, Ghassan S. Kassab, Yiannis S. Chatzizisis

**Affiliations:** a University of Miami, Miller School of Medicine, Center for Digital Cardiovascular Innovations, Division of Cardiovascular Medicine, Miami, Florida, United States; b The University of Texas San Antonio, Department of Mechanical Engineering, Vascular Biomechanics and Biofluids, San Antonio, Texas, United States; c California Medical Innovation Institute, San Diego, California, United States

**Keywords:** optical coherence tomography, image segmentation, nonuniform rational B-spline, three-dimensional reconstruction

## Abstract

**Purpose:**

Optical coherence tomography has emerged as an important intracoronary imaging technique for coronary artery disease diagnosis as it produces high-resolution cross-sectional images of luminal and plaque morphology. Precise and fast lumen segmentation is essential for efficient OCT morphometric analysis. However, due to the presence of various image artifacts, including side branches, luminal blood artifacts, and complicated lesions, this remains a challenging task.

**Approach:**

Our research study proposes a rapid automatic segmentation method that utilizes nonuniform rational B-spline to connect limited pixel points and identify the edges of the OCT lumen. The proposed method suppresses image noise and accurately extracts the lumen border with a high correlation to ground truth images based on the area, minimal diameter, and maximal diameter.

**Results:**

We evaluated the method using 3300 OCT frames from 10 patients and found that it achieved favorable results. The average time taken for automatic segmentation by the proposed method is 0.17 s per frame. Additionally, the proposed method includes seamless vessel reconstruction following the lumen segmentation.

**Conclusions:**

The developed automated system provides an accurate, efficient, robust, and user-friendly platform for coronary lumen segmentation and reconstruction, which can pave the way for improved assessment of the coronary artery lumen morphology.

## 1 Introduction

Optical coherence tomography (OCT) is an intravascular imaging technique that can play a significant role in the planning and optimization of percutaneous coronary interventions.^1^ OCT produces high-contrast, high-resolution cross-sectional images of the coronary arteries using near-infrared rays, which can clearly differentiate the borders between the lumen and vessel wall.^2^ Cardiologists utilizing OCT in the cardiac catheterization laboratory require rapid generation of three-dimensional (3D) lumen models reconstructed from the segmented images for visualization purposes. The manual segmentation of OCT images is a laborious and time-consuming process and requires expertise in OCT imaging segmentation. Therefore, automatic techniques have been developed that can significantly amplify the segmentation speed while maintaining accuracy.^3^^,^^4^ Prior studies have explored several techniques to enhance the automatic OCT lumen segmentation speed and accuracy. One study employed multiple canny edge detection algorithms to detect lumen edges and concentrated on the contour extraction of stented images.^5^ Another study proposed a preprocessing and segmentation method for OCT lumen images that involves conversion between polar and Cartesian coordinates, noise removal using median and Gaussian blur algorithms, and interpolation to connect the disconnected regions of the lumen.^6^ Although one study suggested a smoothly varying threshold approach,^7^ some studies utilized a global threshold approach or a global Otsu threshold derived from performing a one-level decomposition of the preprocessed images.^8^^–^^10^ Recent advances in automated segmentation techniques have utilized machine learning to investigate OCT lumen segmentation.^11^^,^^12^ The success of these automated techniques has been limited by the paucity of appropriate computational and training data resources, however, that can provide results within a suitable time frame and cost efficiency. Furthermore, most of the existing methods have been applicable only to good-quality images without bifurcations^9^ or artifacts,^8^^,^^10^^,^^11^ and their processing times have not been instantaneous (about 1 min per frame).

The existing methods of automated OCT lumen segmentation may be improved in several key areas, including: (i) faster segmentation speed and shorter sample training times (if machine learning is employed); (ii) reliable segmentation in the presence of image artifacts; and (iii) direct 3D vessel reconstruction. To address these limitations, this paper proposes a fully automated approach to OCT lumen segmentation supplemented by manual correction and 3D vessel reconstruction. Specifically, manual correction is necessary for OCT frames that exhibit large bifurcations or significant image artifacts. Our primary objective was to develop a user-friendly, time-efficient, and accurate automated platform for coronary lumen segmentation. Additionally, this tool is intended to facilitate quick and precise 3D reconstruction of the coronary artery lumen.

## 2 Materials and Methods

All procedures were conducted in compliance with relevant guidelines and regulations. The OCT data and angiograms used in this study were obtained from a clinical trial named PROPOT (Randomized Trial of the Proximal Optimization Technique in Coronary Bifurcation Lesions). The study was reviewed and approved by the ethics committee of Teikyo University (IRB Approval No. 15-159-2), and informed consent was obtained from all participants.

### 2.1 OCT Data Collection and Manual Segmentation

Ten clinical cases were included in this study, and OCT images of coronary vessels were acquired using the OPTIS Integrated System (Abbott, Chicago, Illinois, United States). The OCT catheter (Dragonfly, Optis Imaging Catheter) was inserted through a 6F guiding catheter and pulled back at a speed of either 18 or 36  mm/s. Manual lumen segmentation was performed by medical professionals using EchoPlaque 4.0 (INDEC Medical Systems, Los Altos, California, United States) to obtain ground truth data.

### 2.2 Proposed Segmentation and Reconstruction Platform

The platform’s development involved utilizing Rhinoceros 3D, a widely employed computer-aided design software (Robert McNeel & Associates, Seattle, Washington, United States). The OCT frames were imported and visualized in Rhinoceros using a Grasshopper 3D plug-in, a visual programming language and environment.^13^ The custom Python codes for image preprocessing and segmentation were integrated into the Grasshopper 3D environment. The preprocessing of OCT images was performed using Python image packages, such as OpenCV, Skimage, and SciPy. Following the image segmentation process, the lumen was reconstructed in 3D using Rhinoceros.

### 2.3 OCT Image Preprocessing

**Fig. 1:**
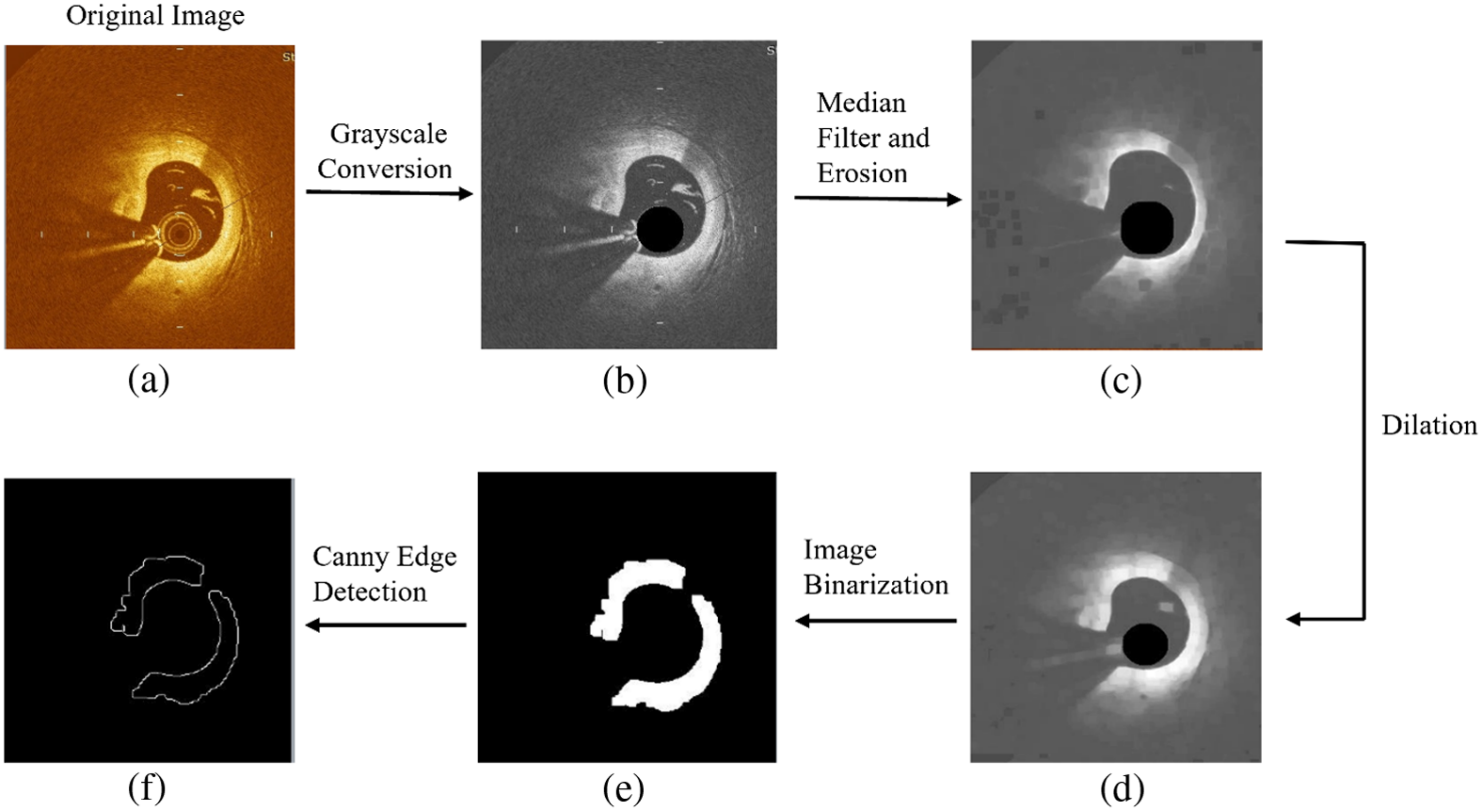
Preprocessing of the OCT images: (a) original image with artifacts, (b) grayscale conversion, (c) median filtered and eroded image, (d) dilated image, (e) binarized image, and (f) Canny edge detection.

In Fig. 1, we present the preprocessing of OCT images. Initially, the original image was converted into a grayscale image, which discarded the color information and retained only the different shades of gray information. Then median filtering was applied, which is a nonlinear technique utilized for reducing speckle noise. The filter selected a pixel from the input image to become the output pixel location at the center of the coordinates in the kernel frames. The median filter eliminated features smaller than the median filter kernel, whereas it had a trivial effect on large fluctuations in intensity or the edges. Following the median filtering, morphological operations were performed.

Morphological processing is another nonlinear image operation that depends on the shape or properties of the morphology. It is based on the relative ordering of pixel values rather than numerical numbers. The morphological analysis of an image utilizes a small template and form, referred to as a structuring element. The structuring element is positioned at each point in the original image and compared with the matching neighborhood of pixels. Certain operations assess whether a component fits inside a neighborhood, and others determine if it intersects or hits the neighboring components. We used grayscale erosion and dilation as the morphological processing techniques in this study.

Grayscale erosion of an image involves assigning the minimum value obtained from the structuring element to each pixel. In this study, the image was convolved with the kernel of an odd-size 5 matrix. Binary erosion of an image f by a structuring element p (denoted f⊖p) resulted in a new binary image g=f⊖p with ones in all locations (x,y) of a structuring element’s origin at which that structuring element p fits the input image f. This means that g(x,y)=1 if p fits f and = 0 otherwise, which is repeated for all pixel coordinates (x,y). Similarly, the dilation of an image was convolved with the kernel of an odd-size 5 matrix. When a structuring element f dilated an image p (denoted f⊕p), the result was a new binary image g=f⊕p with ones in all of the structuring element’s origin locations (x,y), where the structuring element p fits the input image f. Specifically, g(x,y)=1 if p fits f and = 0 otherwise, which is repeated for all pixel coordinates (x,y). In this study, the normalized median value of 164 in the whole range was chosen as the threshold, which we found consistently delivered accurate results in distinguishing the lumen from surrounding tissues and artifacts.

Following the morphological processing, binarization was carried out through rough thresholding. Pixels in the input image with brightness below the threshold level were assigned a value of 0 (black) in the output binary image, and those with brightness above the threshold level were assigned a value of 1 (white). As a result, the final image consisted of only two intensity levels, i.e., 0 and 1. The canny edge detection technique was then applied to refine the image further. The Gaussian filter was employed to suppress the remaining noise, and a double threshold was utilized to identify edges.

### 2.4 Automated Lumen Segmentation, Manual Correction, and 3D Reconstruction of the Lumen

**Fig. 2:**
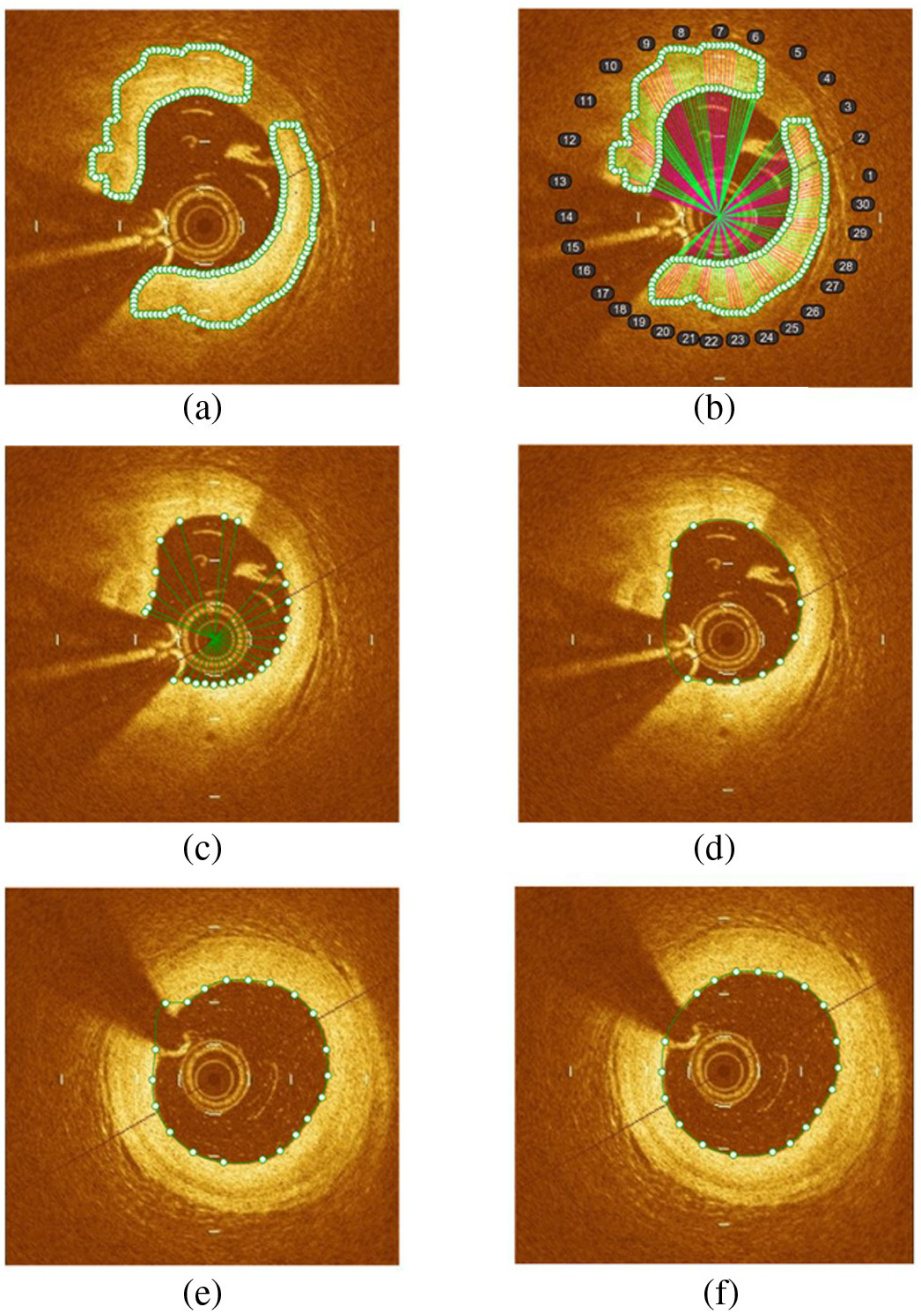
Automatic segmentation method and correction: (a) creation of edge points; (b) all pixel points are connected from the catheter center and are divided into 30 domains based on an axis angle of 12 deg; (c) find the shortest line in each domain; (d) segmented image by connecting the knot points on the image boundary; the green line is the contour estimated by the proposed model; (e) knot point with curvature more than $5\;{\mathrm{mm}}^{-1}$; and (f) curvature corrected image.

The proposed segmentation method utilized nonuniform rational B-spline (NURBS) to represent the lumen shape by connecting limited interpolation (or knot) points on the lumen edge. NURBS offers a high degree of flexibility in representing complex shapes, making it particularly suitable for capturing the intricate contours of the lumen based on the segmentation process. The use of knot points in NURBS allows for easy manipulation and refinement of the shape. This feature was instrumental in our approach as it enabled us to correct the segmentation by simply moving the knot points, ensuring a more accurate representation of the lumen. Furthermore, NURBS can represent smooth curves and surfaces, which is essential for accurately modeling the lumen contours. The process is illustrated in Fig. 2. Initially, all points on the edges were obtained through canny edge detection to determine the appropriate knot points. Next, the catheter center was set as the origin to connect all edge points using straight lines, which were then grouped into 30 domains with evenly anticlockwise 12 deg. The shortest line in each domain was chosen, and the attached edge point became the knot point. Unnecessary knot points were removed based on a set criterion of 0.25 mm, determined through trial and error. If the distance between two-knot points fell within this criterion, one of the points was omitted. Finally, the knot points were connected anticlockwise to obtain the lumen segmentation.

During the segmentation procedure, two additional steps were taken to filter out the interpolation points. First, in domains where the guidewire shadow and branches were present, there were usually few or no edge points (<6), and these points were filtered out to remove the influence of the shadow and branches. Second, if the curvature at a single knot point was too high (more than $5\;{\mathrm{mm}}^{-1}$), this indicated that the knot point might be incorrectly positioned, and it needed to be filtered out.

One of the challenges in automatic segmentation methods is the presence of large branches and abnormal images containing multiple artifacts or image distortions. Previous works have often avoided such frames, but in our method, we chose to manually correct them after the automatic segmentation. We also thoroughly checked all other frames to identify any misplottings, which were also corrected manually. To facilitate the manual correction process, we integrated computer-aided design (CAD) and made NURBS knots easy to control using simple mouse operations. The benefit of this approach is that an expert can correct each frame in just 5 s while ensuring the accuracy of the segmentation.

Following the reconstruction method described in our previous work,^13^ we created a 3D model of the lumen with Rhinoceros using the segmented curves (including corrected ones), which can be further applied for bifurcation reconstruction and analysis. We also reconstructed the 3D lumen models with ground truth segmentation for comparison.

### 2.5 Statistical Analyses

The statistical analyses were conducted using GraphPad Prism 8.0 (GraphPad Inc., San Diego, California, United States), a commercial scientific software designed for creating 2D graphs and performing statistical analyses. Bland–Altman analysis and linear regression were employed to compare the lumen segmentation area, maximum diameter, minimum diameter, and average diameter between the proposed method and the ground truth. A p-value of <0.05 was deemed statistically significant. Frames that underwent manual correction, including both large bifurcation frames and misplotting frames, were excluded from the comparison.

## 3 Results

**Fig. 3:**
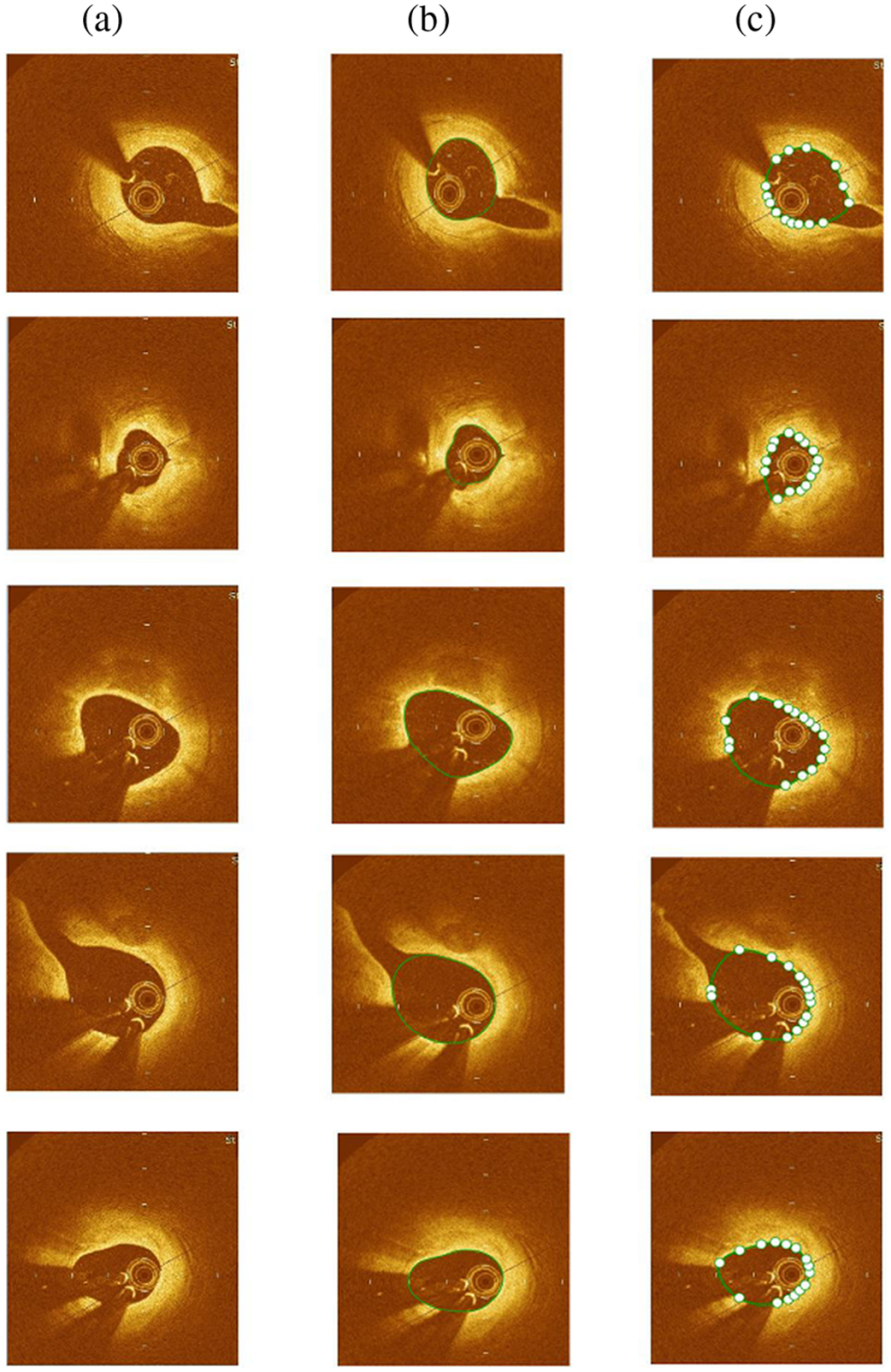
Segmentation for frames with small side branches and guidewire shadows of different sizes. (a) Original OCT images; (b) ground truth manual segmentation; and (c) automatic segmented image by connecting the knot points, and the green line is the contour detection by the proposed model.

**Fig. 4:**
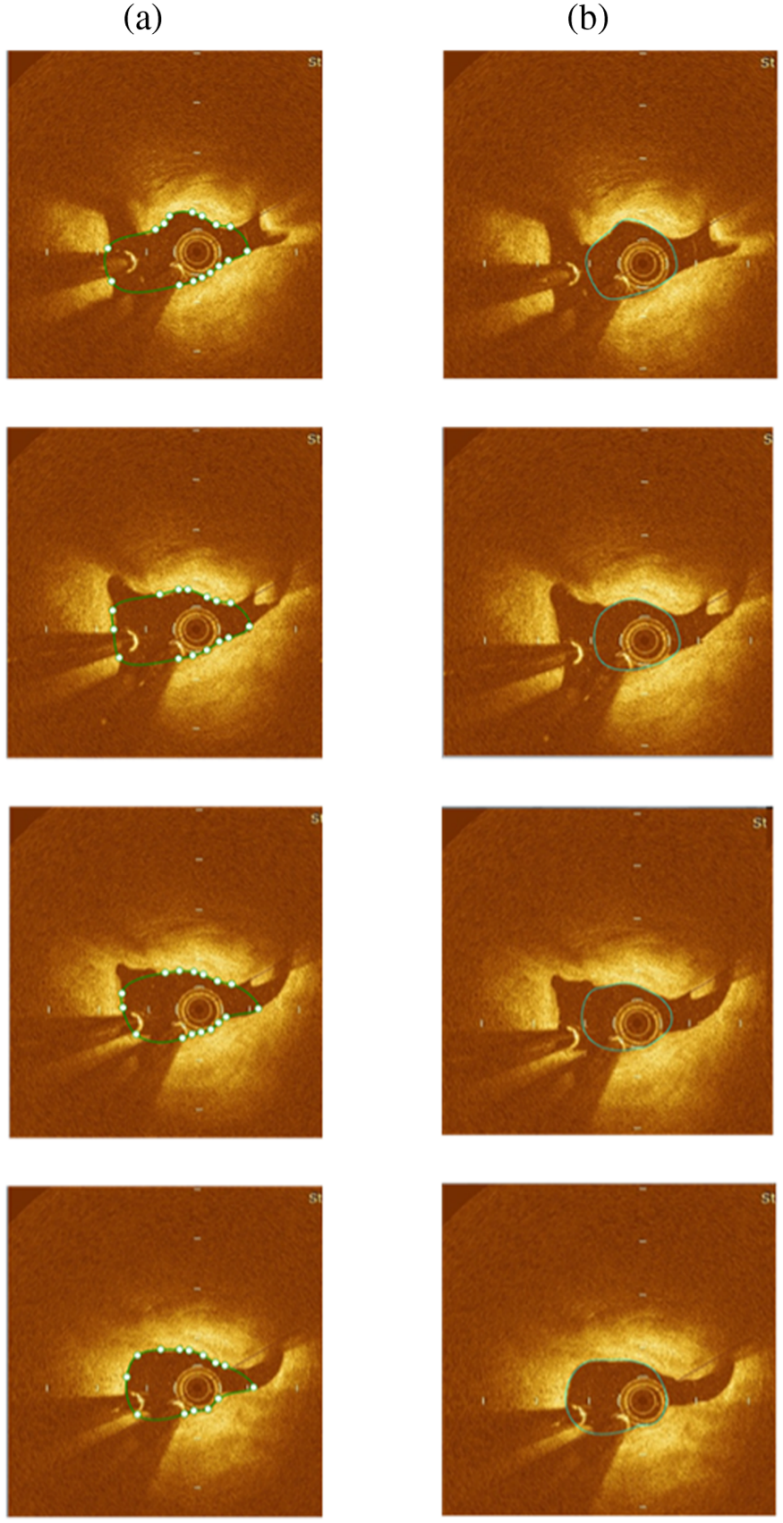
Manual correction after automatic segmentation. (a) Automatic segmentation on bifurcation frames. The green line is the contour detection, and the dots are the pixel points on the boundary. (b) Manual correction on bifurcation frames.

The automated segmentation method was tested on 3300 frames in total, out of which 194 frames had big bifurcations (>3  mm), 121 were abnormal frames with artifacts, and 68 frames had misplottings. Large bifurcations approach very close to the image window size, limiting automation around the edges and requiring manual segmentation. The presence of artifacts, such as residual luminal blood due to suboptimal vessel flushing, which causes signal rich blood swirls in the lumen; blood clots causing complete attenuation of the OCT beam; and the sew-up artifact due to rapid artery or imaging wire movement leading to the lumen border misalignment, lead to significant errors in automation. Additionally, the proposed method encounters challenges in accurately identifying the lumen boundary for certain frames with edge dissections and honeycomb-like appearances due to spontaneous recanalization of thrombi, which lead to misplotting the lumen boundary. Therefore, the remaining 2917 frames were used to validate the automated segmentation method, compared with the corresponding ground truth segmentation performed by clinical specialists. Figure 3 displays the effectiveness of the method in segmenting OCT images with side branches and guidewire shadows of varying widths. In the case of big bifurcation frames, the segmentation results were manually modified using Rhino software to ensure a gradual change in the shape of segmented frames that accurately represents the transition from a branch to the main vessel (Fig. 4). Table 1 shows the time required for automatic segmentation, manual correction in Rhinoceros, and expert segmentation for 10 patients. On average, our method takes 0.17 s per frame for automated segmentation.

**Table 1:** Processing time with the proposed automatic segmentation algorithm and manual segmentation.

		Automated segmentation				Manual segmentation
Case number	Frame number	Time (s)	Manual correction of frames with bifurcation and multiple artifacts (s)	Manual correction of misplottings (s)	Total time (min)	Time (min)
Case 1	430	76	175	0	4.2	138
Case 2	410	72	135	25	3.9	126
Case 3	410	72	145	20	4.0	130
Case 4	230	40	90	0	2.2	70
Case 5	370	65	160	10	3.9	120
Case 6	360	63	175	55	4.9	120
Case 7	235	41	120	35	3.3	130
Case 8	215	38	100	40	3.0	130
Case 9	340	59	175	135	6.2	87
Case 10	300	52	310	40	6.7	84

**Fig. 5:**
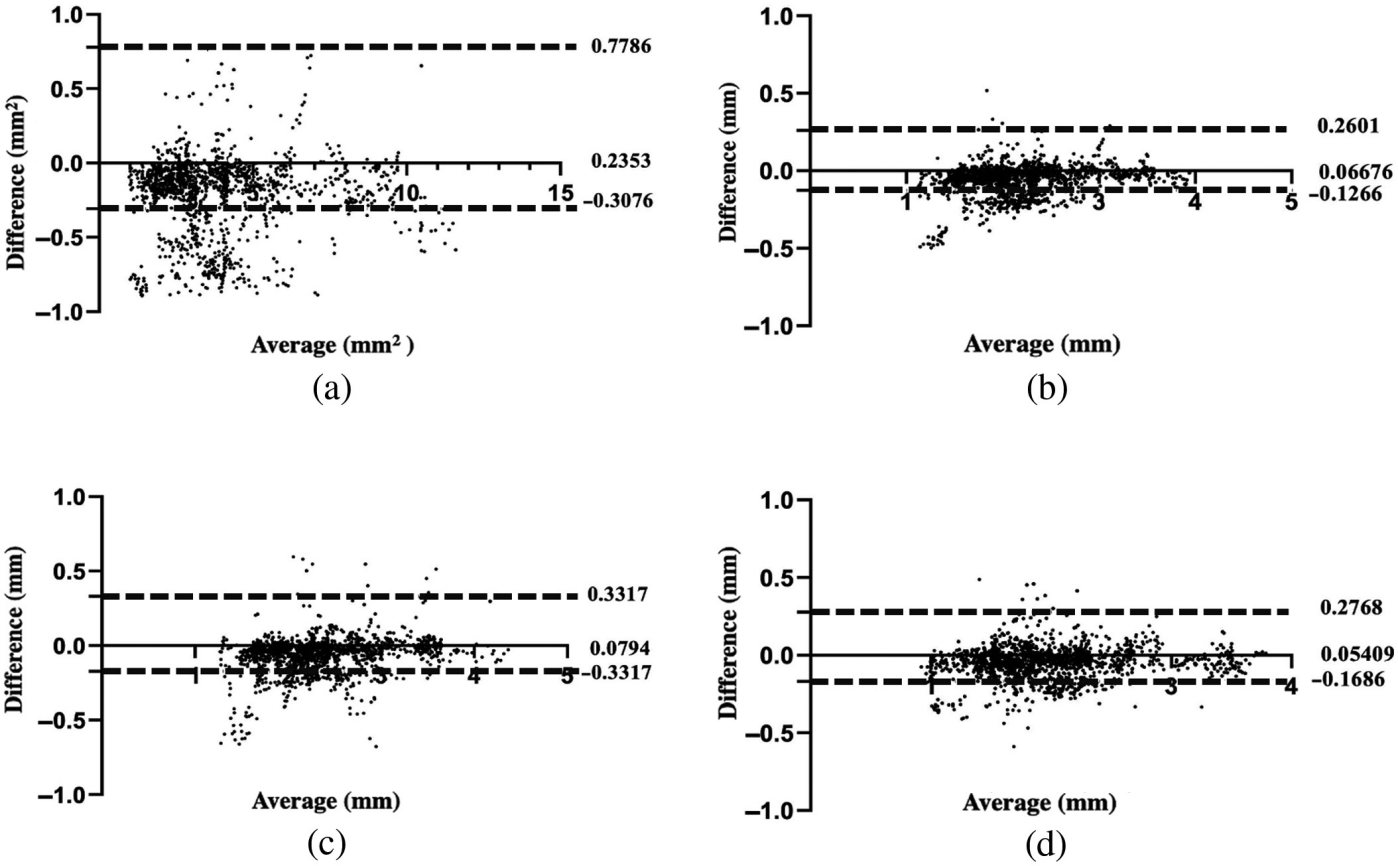
Bland–Altman plots for comparison of the proposed algorithm and manual segmentation: (a) area, (b) average diameter, (c) maximum diameter, and (d) minimum diameter.

The proposed OCT segmentation approach was found to exhibit good agreement with the ground truth results. Specifically, the bias value for the proposed model was $0.24\;{\mathrm{mm}}^{2}$ for the area, 0.07 mm for the average diameter, 0.08 mm for the maximum diameter, and 0.05 mm for the minimum diameter. The 95% limit agreement was calculated for each of these parameters, yielding the following ranges: (−0.31 to $0.78\;{\mathrm{mm}}^{2}$) for the area, (−0.13 to 0.26 mm) for the average diameter, (−0.17 to 0.33 mm) for the maximum diameter, and (−0.17 to 0.28 mm) for the minimum diameter (Fig. 5).

**Fig. 6:**
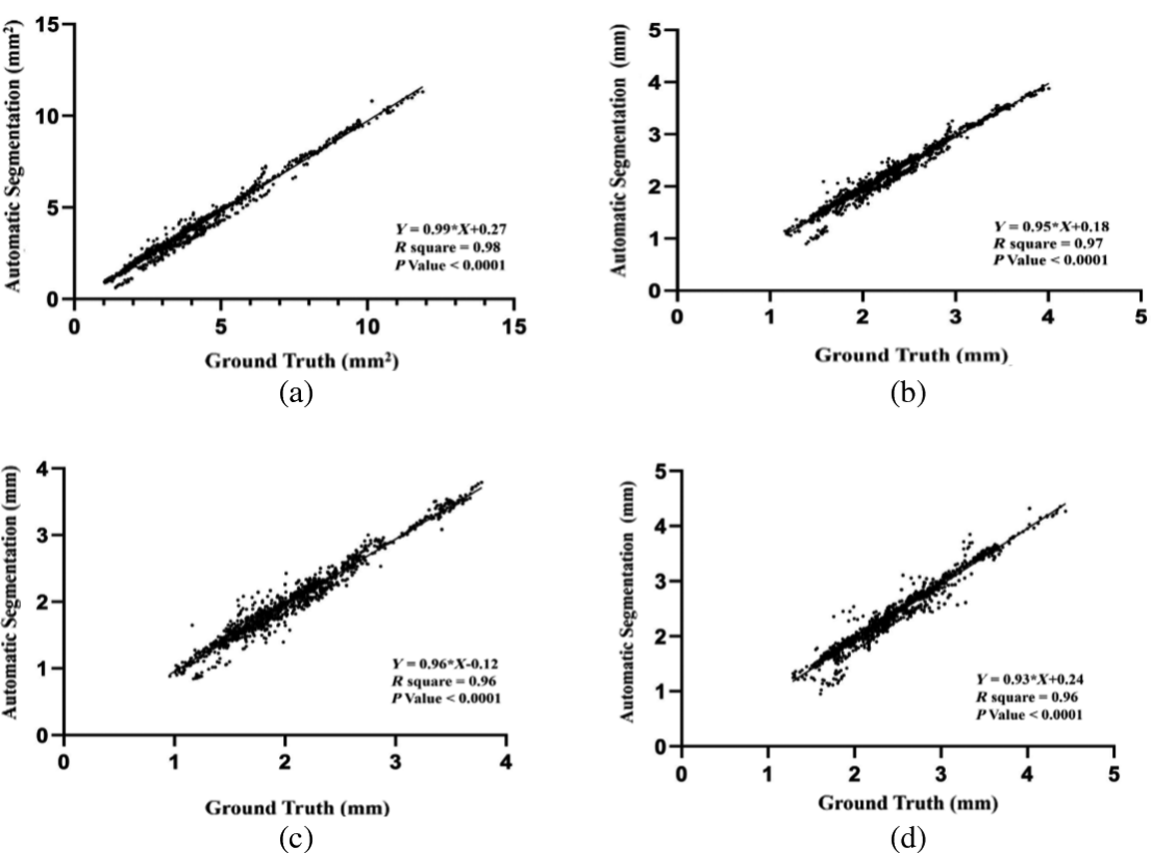
Linear regression analysis of the proposed algorithm and manual segmentation: (a) area, (b) average diameter, (c) maximum diameter, and (d) minimum diameter.

The scatter graphs demonstrated that the proposed automated segmentation was in closer agreement with the ground truth values, as evidenced by the proximity of the data points to the regression line (Fig. 6). The R-squared values for the lumen area, average diameter, maximal diameter, and minimal diameter were 0.98, 0.96, 0.95, and 0.95, respectively. The corresponding equations of the regression lines were as follows: Y=0.99X+0.27 for the lumen area, Y=0.95X+0.18 for the average lumen diameter, Y=0.93X+0.24 for the maximal lumen diameter, and Y=0.96X−0.12 for the minimal lumen diameter. The p-values for the lumen area, average diameter, maximal diameter, and minimal diameter were all found to be <0.001.

**Fig. 7:**
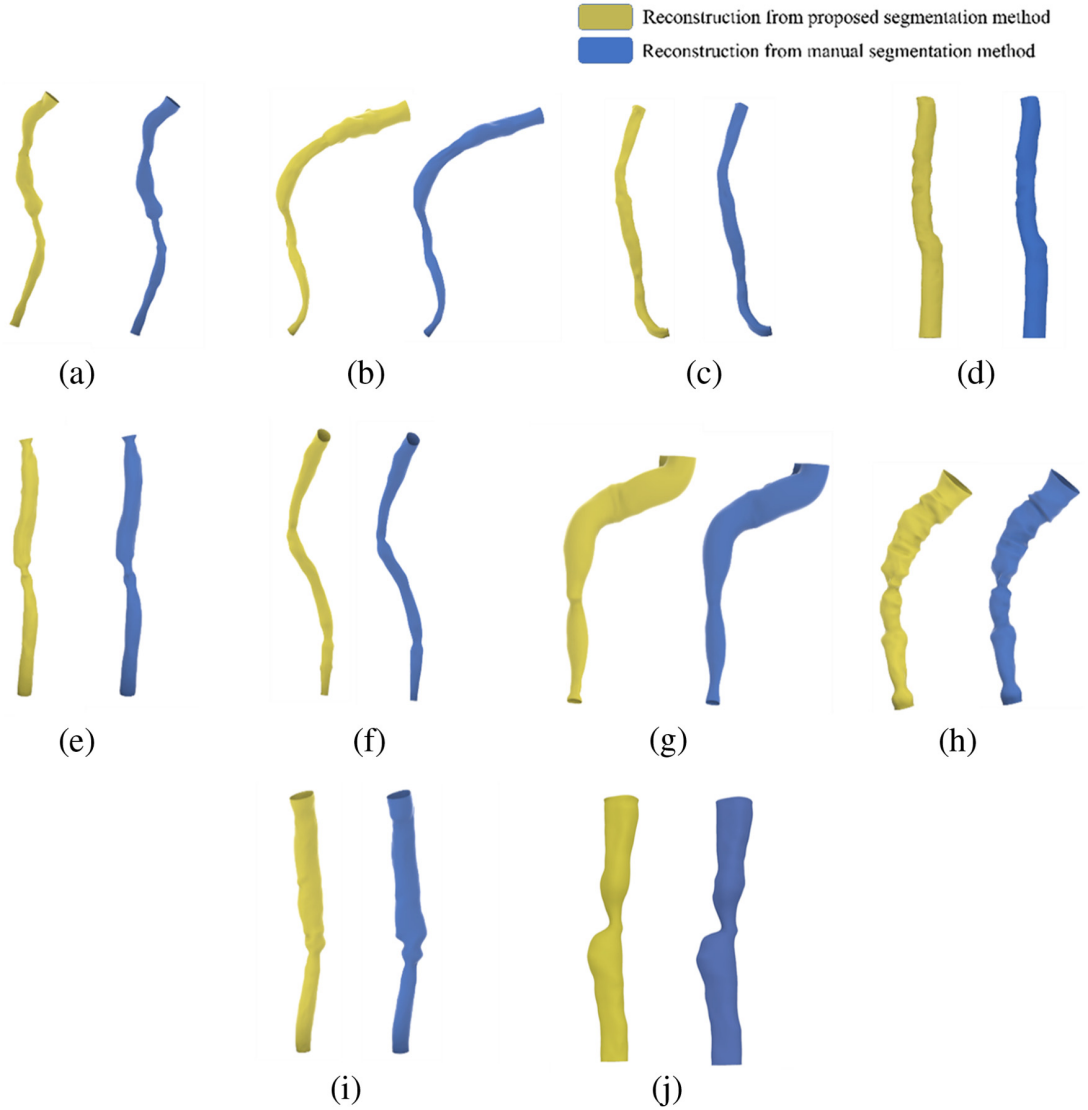
Comparison of the 3D vessel reconstruction between the proposed algorithm (after manual segmentation correction) and the manual segmentation method: (a)–(j) cases 1 to 10.

In the last step of our study, we employed Rhino and Grasshopper to directly reconstruct 3D lumen models using the results of the automatic segmentation (with manual corrections) and compared them with the models generated from the ground truth segmentation. The reconstruction process was found to take ∼6  s per case. The resulting models were found to be fully compatible with the ground truth models, as illustrated in Fig. 7.

## 4 Discussion

We introduce a rapid methodology and platform for automatic OCT lumen segmentation and 3D lumen reconstruction that represents an advancement over the current state-of-the-art methods. Our technique has been demonstrated to be time-efficient, accurate, robust, and user-friendly when applied to clinical data. This approach has the potential to be widely used in the clinical setting to provide highly accurate information about coronary artery lumen in near real time, with 3D reconstructed models that can aid in clinical planning and decision-making in the cardiac catheterization laboratory.

Lumen segmentation is a crucial beginning step in processing OCT images. The accuracy and speed of this process directly influence the subsequent reconstruction and clinical analysis. OCT images are frequently affected by artifacts, guidewire shadows, and branching, however, which makes efficient processing of these images a significant challenge. This necessitates the preprocessing step to obtain a binary image of the intimal layer border to overcome artifacts and improve the image quality for further assessment. However, guidewire shadow is usually regular and constant in size, which makes correction easier. The gaps caused by bifurcations and other large artifacts can have variable shapes, which can cause the shape of the segmented lumen to be irregular. Interpolation of these regions is needed to draw the final lumen contour as close as possible to the expected values. Although several approaches have been proposed to address these difficulties in a time-efficient manner, most of them have processing times of more than 1 s per frame or require extensive training of the model with large datasets. For instance, Pociask et al.^6^ used 4137 images, and their model required 2 to 5 s per frame. Athanasiou et al.^8^ proposed an approach that took 40 s per frame for 556 images, Moraes et al.^10^ required 5.9 s per frame for 290 images, and Macedo et al.^14^ suggested a 15-s processing time per frame for 1328 images. Cheimariotis et al.^15^ proposed an approach using 1812 images that processed each frame in <1  s, and Kim et al.^16^ required an average of 0.367 s per image for 30 images. Tsantis et al.^2^ proposed an approach that required 0.937±0.045  s per image for 2710 images. A comparative study of the automated segmentation approaches of these studies and the processing times is presented in Table 2.

**Table 2:** Comparison of the proposed method with the current state-of-the-art methods.

Authors	Total frames	Time taken	Segmentation accuracy metrics	Limitation
Proposed model	3300	0.174 s per frame	Linear regression r=0.99 (area); 0.95 (mean luminal diameter, MLD); 0.93 (max); 0.96 (min)	1. The method was not tested on images with stents
				2. The method was not applicable for wall and plaque material segmentation
			$R^{2}=0.98$ (area); 0.96 (MLD); 0.95 (max); 0.95 (min)	
			Bias:	
			Area (${\mathrm{mm}}^{2}$) = 0.24 (−0.31 to 0.78)	
			MLD (mm) = 0.77 (−0.13 to 0.26)	
			Maximal (mm) = 0.08 (−0.17 to 0.33)	
			Minimal (mm) = 0.05 (−0.17 to 0.28)	
Pociask et al.^6^	667	1.09 s (average) per frame	Automation mean versus ground truth mean; absolute difference:	The strategy was not tested on images containing stents
			Lumen area (${\mathrm{mm}}^{2}$) = 5.99 (5.83 to 6.14) versus 5.89 (5.74 to 6.04); 0.10 (0.06 to 0.13)	
			MLD (mm) = 2.72 (2.68 to 2.76) versus 2.68 (2.64 to 2.72); 0.03 (0.02 to 0.04)	
			Minimal (mm) = 2.52 (2.48 to 2.56) versus 2.49 (2.45 to 2.53); 0.03 (0.02 to 0.03)	
			Maximal (mm) = 2.91 (2.87 to 2.96) versus 2.88 (2.84 to 2.92); 0.04 (0.02 to 0.05)	
			Relative difference; intraclass correlation coefficient (95% CI):	
			Lumen area = −1.12% (−1.55% to −0.68%); 0.97 (0.97 to 0.98)	
			MLD = −1.15% (−1.48% to −0.83%); 0.96 (0.95 to 0.97)	
			Minimal = −1.11% (−1.44% to −0.78%); 0.98 (0.98 to 0.98)	
			Maximal = −0.81% (−1.23% to −0.39%); 0.91 (0.89 to 0.92)	
Athanasiou et al.^8^	556	40 s per frame	Average overlap ratio: 0.99; nonoverlapping area ratio: 0.02, between automation and manual segmentation for all OCT images	1. Although the technique could detect the lumen boundary in images with a small quantity of residual blood or small thrombi, it could not offer an accurate estimate of the plaque composition in these segments
			Bland–Altman (area) limits of agreement: $-0.080\pm 1.96\times 0.082\;{\mathrm{mm}}^{2}$	
				2. The algorithm was trained using a small dataset of 60 images
				3. The algorithm was not tested on bifurcation images
			Pearson correlation coefficient = 0.99	
			Positive predictive value (PPV) = 0.98	
Macedo et al.^14^	1328	15 s per frame	Automated analysis versus manual segmentation	1. The method was not tested on images with stents
			Minimum lumen area (MLA):	2. The method could not analyze images with substantial luminal blood
			Nonbifurcation (NBR) without $\text{correction}=5.6\pm 3.1\;{\mathrm{mm}}^{2}$ versus $5.4\pm 3.0\;{\mathrm{mm}}^{2}$; mean $\text{difference}=0.19\pm 0.{13\;\mathrm{mm}}^{2}$	
			Bifurcation (BR) without $\text{correction}=6.4\pm 2.5\;{\mathrm{mm}}^{2}$ versus $5.3\pm 2.3\;{\mathrm{mm}}^{2}$; mean $\text{difference}=1.2\pm 0.83\;{\mathrm{mm}}^{2}$	
			Bifurcation with correction $(\mathrm{BRC})=5.1\pm 2.2\;{\mathrm{mm}}^{2}$ versus $5.3\pm 2.2\;{\mathrm{mm}}^{2}$; $\text{mean difference}=0.52\pm 0.{81\;\mathrm{mm}}^{2}$	
			Dice similarity index (DSI):	
			NBR = 97.3 ± 1.5	
			BR = 88.3 ± 8.4	
			BRC = 90.5 ± 9.7	
			Volume overlap error:	
			NBR = 94.7 ± 2.7	
			BR = 79.9 ± 12.4	
			BRC = 83.8 ± 13.6	
			Hausdorff distance (H):	
			NBR = 0.15 ± 0.09	
			BR = 0.97 ± 0.54	
			BRC = 0.41 ± 0.37	
			RMS symmetric surface distance:	
			NBR = 0.04 ± 0.03	
			BR = 0.35 ± 0.23	
			BRC = 0.14 ± 0.12	
			Linear regression (area) NBR = 0.12, $R^{2}=0.998$	
Cheimariotis et al.^15^	1812	<1 s per frame <60 s for 100 frames	Regression analysis r=0.99	If the number of images input to the algorithm is 2, a process called 3D smoothing is performed, which prolongs the segmentation time
			Dice sensitivity index = 0.935 (stented), 0.925 (nonstented)	
			$R^{2}$ (area) = 0.97 (stented), 0.92 (nonstented)	
Kim et al.^16^	30	0.367 ± 0.005 s (average) per frame	Automation mean versus ground truth mean:	Misdetections around guide wire shadow neighbors were in several of the generated images
			True positive area fraction = 99.21 ± 0.51	
			False positive area fraction = 0.30 ± 0.15	
			False negative area fraction = 0.79 ± 0.51	
			Maximum false positive deviation = 0.15 ± 0.03	
			Maximum false negative deviation = 0.11 ± 0.07	
			Sensitivity (%) = 99.7%	
			Specificity (%) = 99.2%	
Tsantis et al.^2^	2710	0.937 ± 0.045 s (average) per frame	Average overlap of 0.937 ± 0.045 between automation and manual segmentation for all OCT images	In several frames where the lumen boundaries were partially invisible, it was not possible for the algorithm to approximate the vessel lumen border
			Maximum lumen diameter ranged from 3.28 to 4.80 mm	
			Overlap (DSI) = 0.937 ± 0.045	
Balaji et al.^17^	12,011	0.095 s (average)	Dice score = 97.31± 4.52	1. The time taken for training the dataset was 3 h and 40 min
			Sensitivity (%) = 95.05 ± 6.69	
			Specificity (%) = 99.66± 0.56	2. The model ignored bifurcations and segmented the lumen in the images with bifurcations based on interpolation from the previous frames
			Hausdorff distance (H)=3.30±1.51 μm	
				3. The frequency of suboptimal segmentation was higher compared with other comparison algorithms
Moraes et al.^10^	290	5.9 ± 3 s per frame	Automation mean versus ground truth mean:	1. The proposed method was based on the older time-domain OCT technology. Its application in the present-day Fourier-Domain OCT technology (FD-OCT) was not studied
				2. The study only performed binary morphological reconstruction of vessels. 3D reconstruction of the vessel was not possible using the proposed technique
			True positive area fraction = 99.29 ± 2.96	
			False positive area fraction = 3.69 ± 2.88	
			False negative area fraction = 0.71 ± 2.96	
			Maximum false positive deviation = 0.10 ± 0.07	
			Maximum false negative	
			deviation = 0.06 ± 0.10	
			Overlap ratio = 95.4% ± 4.8	
			Overlap Dice = 97.8% ± 2.16	
Sihan et al.^5^	4137	2 to 5 s per frame	MLA on automated $\text{analysis}=5.1\pm 2.2\;{\mathrm{mm}}^{2}$ versus manual $\text{segmentation}=5.0\pm 2.2\;{\mathrm{mm}}^{2}$	Because the number of instances in this study was restricted, a larger number of cases is needed to determine whether the good score of totally automated detection in 97% of the images can be maintained for larger populations
			Relative difference = 0.4% ±1.8%	
			Regression analysis r=0.99	
Zhao et al.^18^	268	N/A	Accuracy = 99.66% ± 0.25	Small sample size
			Dice coefficient (DICE) = 99.32% ± 0.58	
			Jaccard index (JS) = 99.40% ± 0.42	
			Hausdorff distance (H) = 0.06 ±0.52	
			Linear regression analysis r = 0.994	
			$R^{2}=0.991$	
Akbar et al.^19^	5931	176 s	Linear regression analysis r=0.988	Not applicable on stented vessels
			Overlapping ratio = 0.931	
			Nonoverlapping area ratio = 0.101	
Cao et al.^20^	4618	N/A	True positive rate = 0.83	Unsuitable for complicated structures (like trifurcation)
			True negative rate = 0.99	
			PPV = 86.8%	
			Negative predictive value = 98.7%	
			Main vessel Dice similarity index = 0.96	
			Side branch Dice similarity index = 0.78	

The ideal method for OCT lumen segmentation of coronary vessels should exhibit two essential characteristics: (i) accurate identification of lumen boundaries and (ii) rapid processing speed with minimal human involvement. Our proposed automatic lumen segmentation technique achieves a processing time of 0.17 s per frame, with an additional 5 s required for manual correction for each frame in the bifurcation region. Based on our knowledge, our approach demonstrates one of the fastest image processing speeds while delivering comparable accuracy to other existing methods.

Our methodology’s efficient and precise processing can be attributed to two main factors. First, we have established that a NURBS curve passing through limited knot points can accurately represent the shape of the lumen. This fact has been validated through extensive manual segmentation operations conducted in our previous works.^13^^,^^21^ The algorithm employed to determine these knot points after image preprocession is straightforward and robust as it involves only line creation, domain division, and length comparison. The filtering methods for knot points apply simple geometrical comparisons, such as point distance and curvature. As a result, our method extracts the essential characteristics of the lumen shape rapidly and accurately without complicated machine learning techniques and coordinate system transformations. By contrast, other works have relied on more complex machine learning methods with varying degrees of success. For example, Balaji et al.^17^ proposed a deep learning model for segmenting OCT images that required training the dataset for 3 h and 40 min, which is a significant limitation compared with our proposed model. Similarly, Guo et al.,^12^ Kerkeni et al.,^22^ Abdolmanafi et al.,^11^ and Macedo et al.^23^ used supervised machine learning techniques, such as support vector machines and least squares regression, to segment OCT data. Although these attempts achieved some success, they were often hampered by a lack of training data and the intrinsic limitations of coronary artery OCT. More recently, convolutional neural networks and linear regression algorithms have been used to designate lumens using a points technique, as opposed to successful pixel-wise segmentation.^24^ However, training machine learning models is challenging, particularly in the medical imaging field due to the exponential increase in the number of trainable parameters as the network depth increases. This can result in extremely lengthy training times and require complex hyperparameter adjustment approaches, particularly when dealing with high-resolution images, often 512×512  pixels or larger.

Second, our method integrates CAD software (Rhinoceros), which offers a great convenience for manual correction and seamless data transfer from segmentation to reconstruction. This contrasts with other works that often use a third-party code for manual correction and reconstruction. Our method can provide a reconstructed vessel in real time, which is a significant advantage over other methods.^25^^–^^27^

Accurate segmentation and reconstruction of the bifurcation region of a vessel is a challenging task for any methodology as the merging of a branch into the main vessel leads to a continuous change in the vessel’s shape and lumen contour. Thus accurately segmenting the gradually changing bifurcation frames requires careful manual effort to achieve a reasonably accurate transition from a side branch to the main vessel. Macedo et al.^14^ addressed this issue by initially performing automatic segmentation of bifurcations with significant side branches, followed by validation of the automated segmentation with manual segmentation of the same frame by an expert, which served as the ground truth. In the proposed approach, bifurcation frames were automatically segmented and then corrected manually by an expert to achieve the desired vessel shape and ensure a more precise reconstruction.

We performed a comprehensive validation of our methodology by comparing it to ground truth data provided by medical experts. Our validation process involved the use of various dimensional indices, including lumen area and lumen maximum, minimum, and average diameters. We found that our proposed OCT segmentation approach produced results that were in good agreement with the ground truth data. Specifically, we obtained $R^{2}$ values of 0.98, 0.97, 0.96, and 0.96 for lumen areas, average diameters, maximum diameters, and minimum diameters, respectively. The p-values for these measurements were all below 0.0001, indicating strong statistical significance.

Our methodology offers a user-friendly feature due to the visual programming language tool, Grasshopper 3D. In contrast to conventional text-based codes, such as MATLAB, Grasshopper 3D facilitates a modularized workflow, even for operators without programming experience. This tool requires minimal intervention for the parameter settings. The feedback from clinical physicians, who are end users of the methodology, indicates that the learning curve is minimal and that it allows them to become familiar with the tool quickly. This feature improves the usability of the tool in interpreting the results and allows for manual intervention where necessary. Additionally, it enables users to adjust segmentation parameters with ease and view the results in almost real time. Therefore, our methodology is practically feasible and versatile for different types of real patient data and diseased bifurcation anatomies, regardless of the degree of stenosis.

Our proposed method has significant applications in offline clinical studies. The precise segmentation and reconstruction of the lumen can offer valuable insights for researchers in understanding the scope, severity, and precise vessel anatomy of coronary artery stenosis. This can aid in the personalized planning of stenting techniques for modeling and simulation purposes, leading to a deeper understanding of patient outcomes. Furthermore, extending this methodology to other imaging modalities is a possibility. The fundamental concepts of our methodology, particularly the efficient lumen edge extraction, can provide a valuable foundation for future research seeking to adopt similar techniques to diverse imaging modalities, such as intravascular ultrasound imaging or coronary computed tomography angiography, keeping in mind their own distinct challenges and necessitating tailored approaches for successful adaptation.

## 5 Limitations

There are several limitations to our work. First, our automatic method is not suitable for analyzing large bifurcations, as there may not be sufficient edge points present. Second, lumen shapes in bifurcations can change gradually, further complicating automatic detection. Therefore, manual correction is necessary for such cases. Third, our method focuses only on lumen edge detection, which means that it is unable to recognize the medial wall and characterize the plaque tissues. Fourth, our method was not tested on stented coronary arteries, limiting the usefulness of this methodology for clinical applications. Future studies will focus on testing this method on the stented vessels. Finally, our study used manual segmentation by clinical experts as the gold standard to validate the performance of the proposed methodology. There is a possibility of unreliable estimation by experts, however, due to the difficult segmentation of certain atypical frames. Thus it requires further validation of the proposed methodology using a more accurate reference. Despite these limitations, we believe that our work can serve as a solid foundation for future studies on lumen walls and tissue segmentation.

## 6 Conclusion

We introduced a platform that enables rapid and automated coronary OCT lumen segmentation, with the added convenience of manual correction, followed by a seamless 3D vessel reconstruction process. We evaluated the accuracy of the segmented lumens and reconstructed vessels against ground truth data (covering a wide range of anatomical complexities) and found our method to be accurate and time efficient. Our platform employs NURBS’ distinctive characteristics and is integrated with CAD software and a virtual programming environment, resulting in a time-efficient, accurate, robust, and user-friendly tool for clinical image processing in nearly real time. This platform has the potential to aid clinical planning, education, and decision making for coronary catheterization.

## Data Availability

Python code used in the manuscript can be accessed at the Code Ocean: https://codeocean.com/capsule/7512538/tree.
